# Longevity through immunity: the unusual naked mole-rat immune system

**DOI:** 10.1007/s11357-025-01874-5

**Published:** 2025-09-16

**Authors:** Tanvi T. Patel, Rileigh Rubar, Vince G. Amoroso, Martha A. Delaney, Thomas J. Park, Rochelle Buffenstein

**Affiliations:** 1 Dept of Biological Sciences, University of Illinois, 845 W Taylor St., Chicago, IL 60607 USA; 2 Zoological Pathology Program, College of Veterinary Medicine, University of Illinois Urbana-Champaign, Brookfield, IL 60513 USA

**Keywords:** Thymus; Spleen, Ectopic thymus, NK cells, GdT cells, Myeloid cells, Lactoferrin-high neutrophil

## Abstract

Naked mole-rats (NMR, *Heterocephalus glaber*) defy Gompertzian rules of mortality and age-dependent senescence to lead healthy lives well into their fourth decade. The NMR immune system likely contributes to this phenotype by protecting against infections, regulating tissue repair, and eradicating neoplastic cells. In keeping with their many other paedomorphic traits, adult NMRs retain functional thymic tissue in the submandibular triangle. Despite this additional thymic tissue, perinatal NMR thymi are half the size of mouse thymi, yet both species show similar thymic involution trajectories. Smaller thymi may indicate less diverse thymocyte clonotypes that need to complete selection before being released as functional, self-tolerant T cells. Transcriptomic studies revealed that when compared with mice, NMRs have a larger myeloid to lymphoid ratio, lack natural killer (NK) cells, and that the αβ clonotypic diversity of NMR CD8 T cells is limited and smaller than that of their CD4 T cells. NMRs, strikingly, have a large circulating population of γδ T cells with two distinct subsets; one with a large diversity of γ and δ TCR loci and the other, a cytotoxic T cell subset with a dominant clonotype. Collectively, these data suggest that NMRs, living in a sealed milieu, rely more upon the innate immune response and have evolved under relaxed viral selective pressure. Rather, they have selected for an atypical myeloid-biased mode of innate immune surveillance and circulating populations of cytotoxic γδ T cells. In summary, the NMR appears to have been optimized to provide better immunosurveillance, tissue homeostasis, and protection against cancer rather than protection against unlikely environmental exposure to viral pathogens. These features likely contribute to their prolonged healthspan and exceptional longevity.

**Graphical Abstract:**

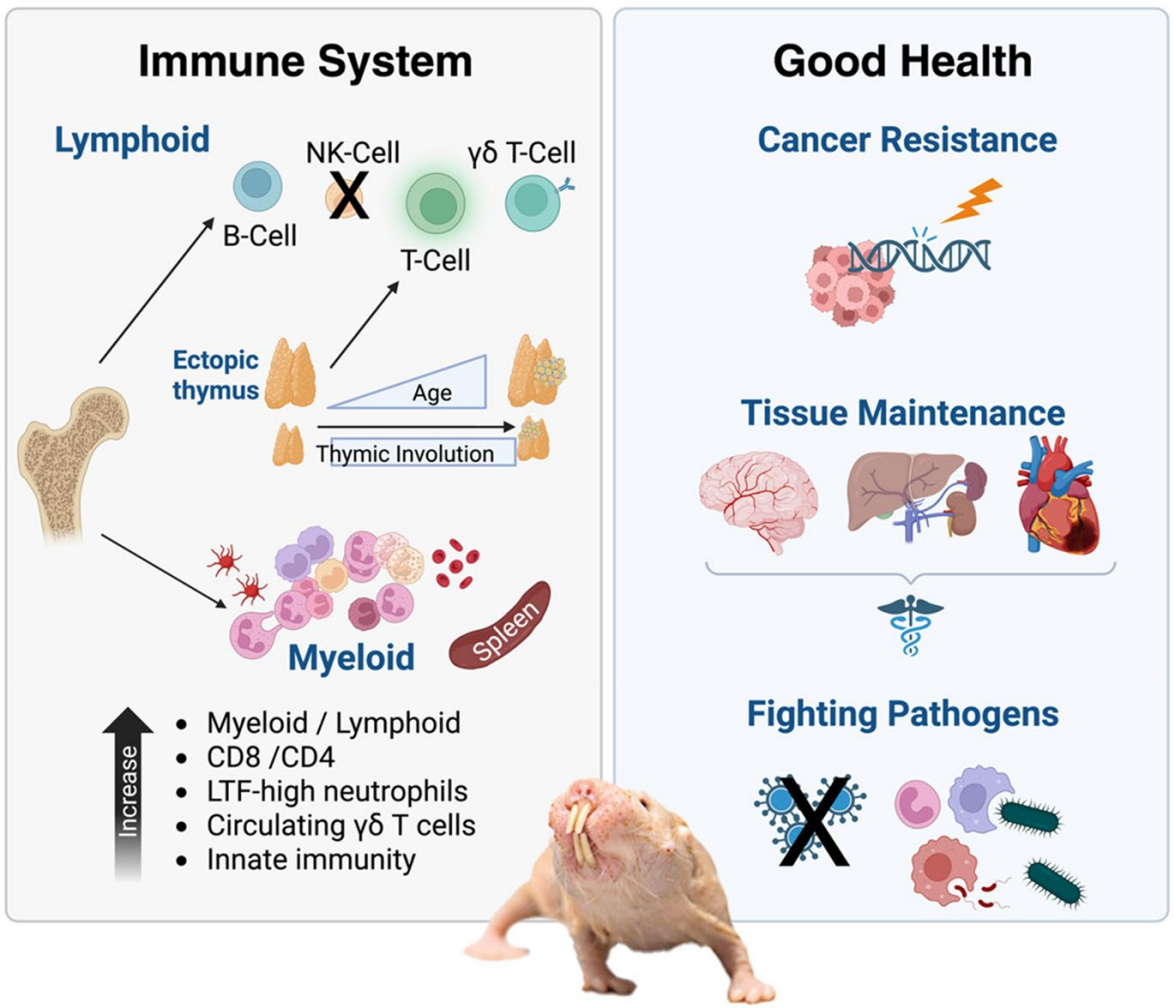

## Introduction

The naked mole-rat (NMR; *Heterocephalus glaber)* has emerged as a robust, naturally evolved, attractive animal model of exceptional biomedical interest [1–3] and has been used as an in vivo model of resistance to inflammatory pain [4, 5], hypoxia [6], hypercapnia [7, 8], aging [2, 9–11], cancer [12–17], infertility [18], neurodegeneration [19–21], and cardiovascular disease [22–24]. NMRs are endemic to the eastern horn of Africa, where they inhabit an extremely harsh, hot, crowded, and resource-restricted underground milieu. They Live in colonies of up to 300 individuals with a strict dominance hierarchy culminating in the presence of a single dominant breeding female. Moreover, they exhibit unique behavioral, physiological, and metabolic traits that are considered highly specialized for the environmental conditions they routinely encounter [1]. Resting communally in deep nests of their below-ground, sealed burrow system, NMRs are extremely tolerant of both hypoxia and hypercapnia and respond to the lower oxygen atmospheres by lowering their metabolic rate and using alternative energy-generating pathways such as fructose metabolism [6]. Similarly, living in the dark confines of a maze-like burrow system, they cannot rely on visual cues but rather use touch, hearing, the sensation of vibrations, and smell to negotiate their abode and stratified social interactions [25].

The NMR exhibits extraordinary longevity for a small (wild-caught weight 33.9 ± 3.4 g [26, 27]; captive adult weight range 25–100 g) Mammal, exceeding 40 years, living an order of magnitude longer than laboratory mice (*Mus musculus*) and rats (*Rattus norvegicus*) [9]. It is the only species known to date that defies Gompertzian laws of mortality; death is stochastic and independent of chronological age [28]. Indeed, the risk of dying across the entire spectrum of observed lifespans is unchanged, even at ages many-fold greater than their allometrically predicted maximum lifespan [28, 29]. Moreover, physiological and molecular studies reveal well-maintained function and tissue homeostasis over more than three decades of life [30]. Relative to other mammals, NMRs exhibit a low incidence of age-related, pro-inflammatory, non-infectious chronic, or degenerative diseases such as arthritis and neurodegeneration [12, 22]. As such, the NMR stands out as an exemplary mammalian model of successful healthy aging.

While NMRs show normal nocifensive responses to pain induced mechanically or by heat, unlike most other animals, they show diminished pain from inflammation and also show insensitivity to painful stimuli such as the burn from capsaicin or the sting from acid [4, 31], the latter features attributed to mutations in voltage-gated ion channels (Na_v_ 1.7), reduced nerve growth factor (NGF) signaling impairing activation of sensory neurons [32, 33], and lack of the neurotransmitter substance P [4]. The mitigated inflammatory pain response to noxious stimuli may impact the activation of the NMR immune response, especially when confronted with chronic systemic conditions. In our quest to elucidate how NMRs defy the aging process and can show negligible senescence, we hypothesize that the NMR has evolved a highly efficient and well-controlled immune system that better regulates tissue repair, protects against infection or noxious agents, and efficiently eliminates cancerous cells. Thereby the NMR immune system has a critical role in the maintenance of organ homeostasis, and as a result thereof in their prolonged good health and lifespan.

There have been only a handful of studies that have examined the NMR immune system, even though the immune response, together with its inflammatory component, likely may play a pivotal role in their unusual ecophysiology [30] as well as their non-cell-autonomous cancer resistance [16, 34]. The paucity of immune studies is primarily because, until recently, there were very few appropriate reagents available, with the NMR tissues showing a lack of cross-reactivity with most antibodies used in human and mouse immunological studies [35, 36]. Most of the recent studies have circumvented these problems and relied primarily on genomic and transcriptomic resources [37–41]. As a result, several unique features of the NMR immune system have recently been elucidated that likely contribute to their extraordinary biology [1].

## The immune system

**Fig. 1:**
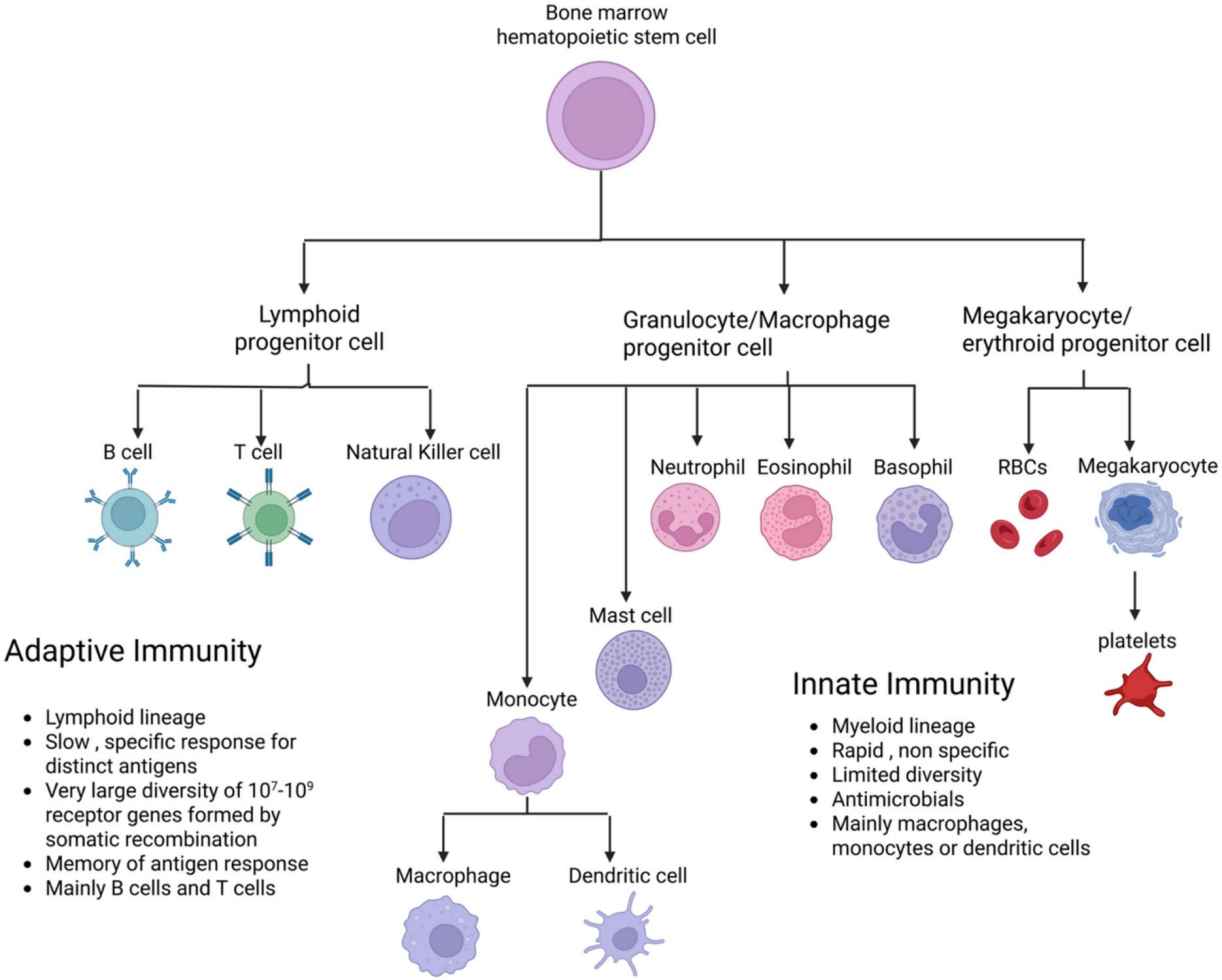
Hematopoiesis and immune cells of mammals and their role in adaptive and innate immunity. Modified from Orkin and Zon 2008 [119] and Lin and Buffenstein (2021) [69]. RBC’s- red blood cells. Figure drawn with BioRender

The immune system helps an organism identify and protect itself from anything it recognizes as stressed or foreign, including mutated cells, toxins, or pathogens. It does this by activating innate and adaptive immune cells through inflammatory signaling to facilitate their eradication and restore homeostasis (Fig. 1). The first line of defense is the physical barriers preventing foreign bodies or pathogens from entry. If these are breached, the next line of defense is hard-wired, non-specific, and rapid. It involves the innate immune system, whereby myeloid (macrophages, monocytes, granulocytes) and lymphoid (natural killer [NK]) cells are recruited to the damaged or pathogen-infected site and promote chemotaxis, directing the migration of other immune cells (e.g., neutrophils and T cells) to the site of infection, using chemical attractants released by the infected tissue (Fig. 1). The macrophages and other antigen-presenting cells (APCs) are also involved in activating the adaptive immune defense through a cascade of signaling cytokines and antigen presentation, recruiting B and T cells. These lymphoid cells sense infections by integrating the various signals (i.e., by antigens) from cell surface receptors. This slower, more targeted, and specific line of defense enables one to distinguish foreign material from host cells and provides specificity, diversity, and memory to better protect from future attacks from the same pathogen, allergen, or tumor cells. As such, the immune system and its critical inflammatory signaling play a crucial role in both healthspan and longevity, influencing the susceptibility to acute and chronic diseases [42, 43].

Inflammation is a protective reaction to endogenous and external harmful stimuli [44] and is involved in recruiting an appropriate immune response. Unregulated or chronic inflammation is deleterious and may lead to autoimmune diseases (e.g., lupus), as well as localized ailments such as asthma, inflammatory bowel disease, and neuroinflammation. Chronic inflammation also contributes to numerous age-associated “lifestyle” diseases (e.g., diabetes, rheumatoid arthritis, cardiovascular disease) and disabilities. Not surprisingly, chronic inflammation is considered a pivotal biomarker of aging [45] and is now commonly called “inflammaging.” Indeed, the aging process is characterized by changes in immune cell populations, a decline in anti-inflammatory cytokines, and elevated levels of numerous pro-inflammatory cytokines, with high plasma levels of interleukin-6 regarded as a predictive biomarker of all-cause mortality in elderly humans [46]. In particular, the decline in the T cell population results in less effective immunosurveillance, reduced eradication of damaged, senescent, virus-infected, or neoplastic cells, and a more proinflammatory milieu that further triggers proinflammatory cytokine secretions [47]. Excessive levels of myeloid activity and these proinflammatory cytokines exacerbate thymic degradation, and ultimately, these age-related changes lead to a disruption in tissue homeostasis and a decline in physiological function that, in turn, results in increased morbidity and mortality [48, 49].

While most inflammaging studies focus on humans, chronic inflammation and immunosenescense have also been reported throughout the animal kingdom, including worms, insects, mammals, birds, and reptiles [50–53], and primarily affects the adaptive immune system. In many comparative biology studies, the immune and inflammatory responses are evaluated by treating animals or their blood ex vivo with lipopolysaccharide (LPS or endotoxin). LPS is a component of the cell wall of gram-negative bacteria and is detected by toll-like receptor 4 (TLR4). Upon binding to the TLR4 complex, LPS initiates intracellular signaling that leads to the release of cytokines and chemokines, thereby triggering the host’s immune response [54].

Here, we review what is known about the immune system of the long-lived, cancer and toxin resistant NMR and focus primarily on the cells of lymphoid and myeloid lineage [55].

## Immune organs

We, and others, previously examined components of the naked mole-rat immune system using morphology, immunohistochemistry, fluorescence-activated cell sorting (FACS) technology, and single-cell transcriptomics in the key compartments of the immune system: primary lymphoid organs—thymus and bone marrow and secondary lymphoid organs—blood and spleen [36–41].

### Bone marrow

Bone marrow, the spongy-like tissue found in the core of long bones, contains the hematopoietic stem cells (HSCs) from which all the immune cells and red blood cells (RBCs) originate. NMR femurs are both smaller and paler than those of mice, suggesting that fewer erythrocytes and other cells are contained within, a finding confirmed during single-cell isolation for transcriptomic studies [39, 40]. All known hematopoietic cell types documented in other species were present in NMR bone marrow [36, 39, 40]. Both myeloid and lymphoid cells are differentiated in NMR bone marrow, however the distribution of these cells in NMR peripheral blood is more similar to that of humans than other rodents, with increased circulating myeloid cells and fewer lymphocytes [36, 39, 40]. Intriguingly, the NMR immature neutrophils therein resemble the stab-cell-shaped human immature neutrophils more closely than the ring-shaped neutrophils of conventional laboratory rodents [39, 56].

All erythroid cell types (e.g., erythroblasts, mature erythrocytes, orthochromatic erythrocytes, and reticulocytes) are present in NMR bone marrow. In rodents and humans, erythrocytes, myeloid immune cells, and B cell lymphocytes are released from the bone marrow into the blood as functional cells.

### Thymus

Lymphoid T cells released from the bone marrow complete their proliferation, differentiation, maturation, and selection in specialized compartments within the thymus and thereafter are released into the circulation [57, 58]. Specialized thymic epithelial cells facilitate these developmental processes through interactions with their receptors. Positive and negative selection are critical for the completion of T cell development. Positive selection occurs in the thymus cortex and ensures that T cells recognize MHCs, which leads to the development of CD4 + and CD8 + T cells. Negative selection occurs in the thymus medulla and eliminates those T cells that are strongly reactive to self-antigens, preventing them from attacking the body’s own cells and the occurrence of autoimmunity [57–59]. To date, there are no reports of autoimmune diseases in NMRs.

**Fig. 2:**
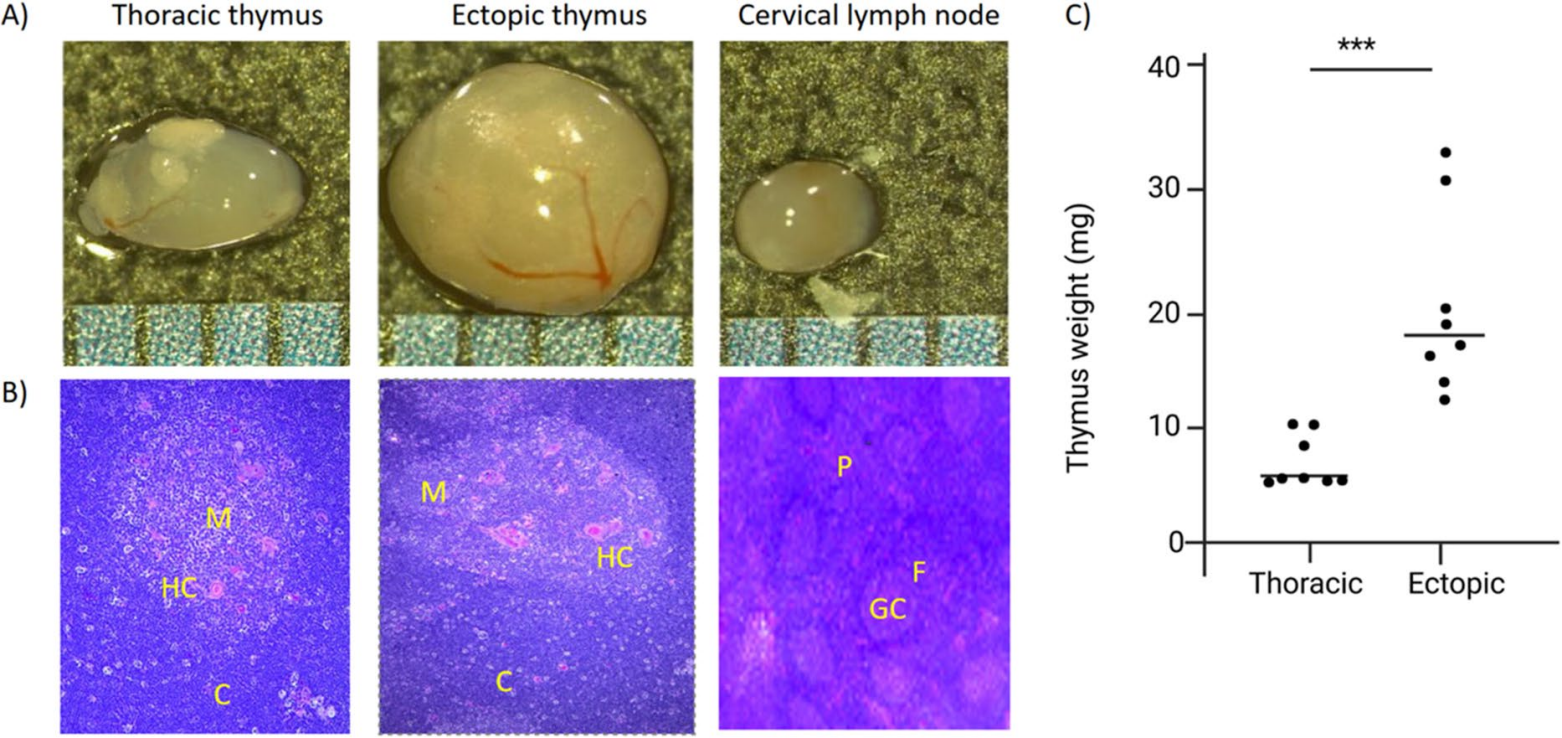
Thoracic thymus, ectopic thymus, and cervical lymph node. **A** The naked mole-rat ectopic thymus, located in the submandibular triangle and is more vascular than cervical lymph nodes. Thymi were placed alongside a ruler, with the Markings representing 0.1inch. **B** Hematoxylin and eosin (H&E) staining of the thoracic and ectopic thymus reveals a similar structure (M = medulla, C = cortex; HC = Hassall’s corpuscle) that is quite different in structure from the cervical lymph node (F = follicle, P = paracortex, GC = germinal center). Images were photographed at the same magnification. **C** The ectopic thymus is approximately four times larger than the thoracic thymus. The thymi from 8 young adult animals were weighed and the median data illustrated by dot plot. Data were compared using paired student t-tests and were significantly different (*p* < 0.01)

**Fig. 3:**
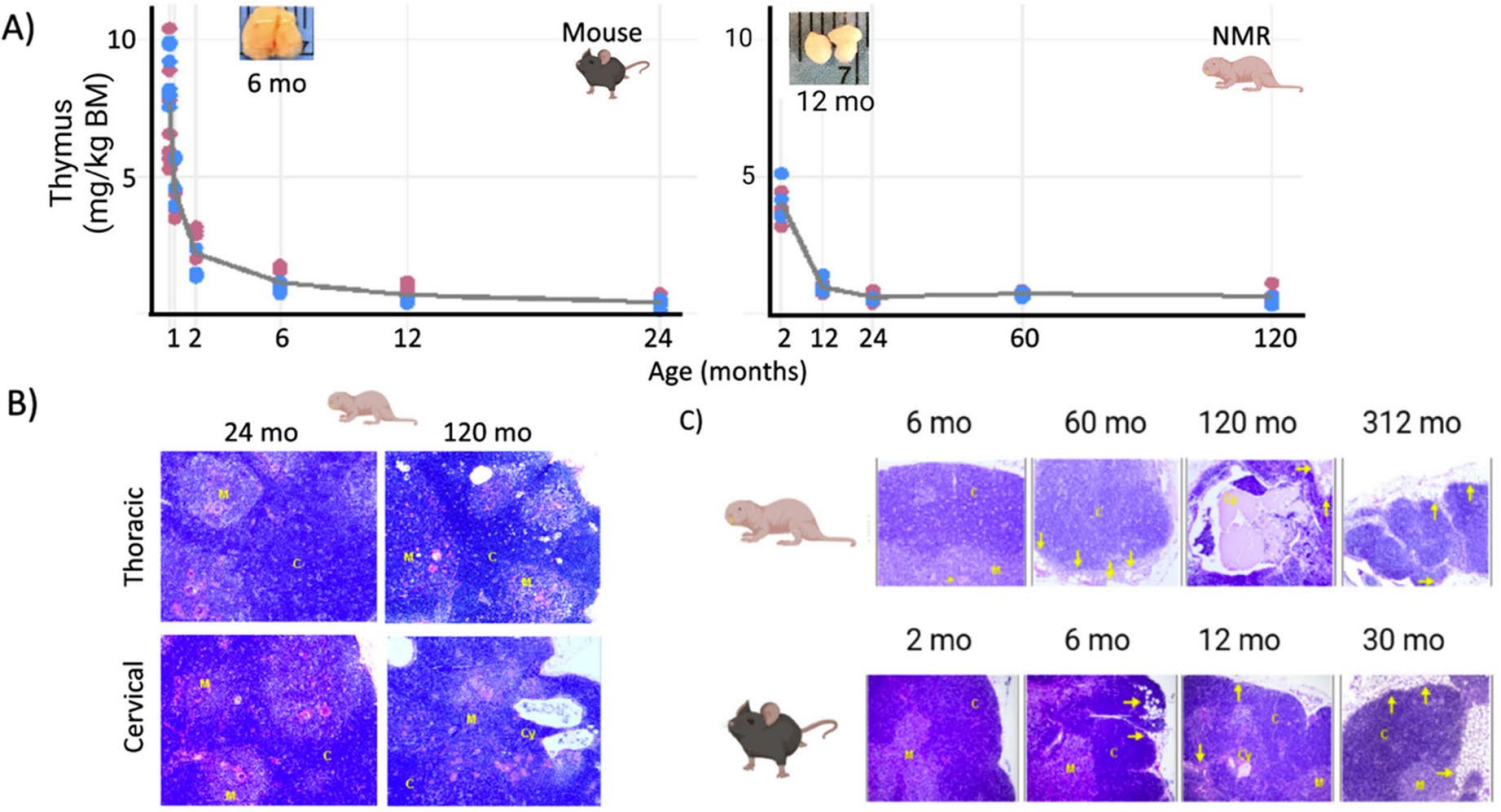
**A** Consistent with relaxed antiviral selective pressure, and therefore anti-viral immunity, early-life naked mole-rat (NMR) thymi are relatively smaller (when expressed per animal body mass (BM)) than those of young mouse thymi. Both species show signs of similar thymic involution with a pronounced reduction in size evident by 2 months of age in mice and by 12 months of age in NMRs. **B** Both the NMR thoracic and ectopic thymi show similar signs of involution when thoracic thymi from two-year-old are compared with those of 10-year-olds. **C** Age-related changes in the thoracic thymus of mice, and NMRs, as seen by H&E histology, show considerable fat infiltration (yellow arrows), lower cell density in both the medulla (M) and cortex (C) and thymic cysts (Cy). Figure modified from [40] and drawn with BioRender

Like other mammals, NMR thymic tissue is found in the thoracic cavity above the heart. Surprisingly, an additional ectopic thymic tissue, identical in cell composition and transcriptome but four-fold larger in size than the thoracic thymic tissue (Fig. 2), is found in the submandibular triangle region of the neck [38–40]. It can be distinguished from the cervical lymph node that is also located in this region in that it is markedly more vascular (Fig. 2A). Normally, during fetal development, the thymus, which arises from the third pharyngeal pouch, migrates to the superior mediastinum with a final location above the heart in the thoracic cavity [60]. Disruptions during thymus organogenesis and migration may lead to ectopic thymic locations, with a cervical location most commonly found in mice and humans [61–63]. The ectopic thymus may be fully functional and generally is asymptomatic. Its abnormal location is thought to be due to specific gene mutations (e.g., TBX1, CHD7, and FOXD1) disrupting thymus developmental progression. It is unknown why the NMR retains a fully functional, large, bilateral pair of cervical/ectopic thymic organs in addition to the pair in the thoracic location throughout adult life; although it is in keeping with the retention of numerous neotenic and paedomorphic traits throughout life in naked mole-rats [64, 65]. Begay reported that the cervical and thoracic thymic tissues are occasionally seen embedded in thermogenic brown fat rather than white adipose tissue [39], a finding we, too, have observed quite commonly in the cervical thymi. We speculate that this atypical location of thymic tissue in thermogenic brown fat may elicit a stronger febrile response, promoting more rapid T cell release. The combined mass of the canonical thoracic thymus and ectopic cervical thymus tissue is surprisingly significantly smaller both in absolute terms and relative to body size than that of the single site of thymic tissue found in mice (Fig. 3A). Both thymic structures, like that of mice, are multi-lobular, separated by interlobular septa, with many medullary sinuses and no germinal centers, and have numerous Hassall’s corpuscles [39, 40]. These are thought to be essential for regulating the development and maturation of T cells, particularly in the selection of regulatory T cells, and also play a key role in removing apoptotic thymocytes [66]. Moreover, both thymic tissues are CD34 + and have more basophilic cytoplasm than observed in lymph nodes [38]. Unlike mice, NMR thymic tissues have a lower cell density, with unclear demarcation between the cortex and medullary regions (Fig. 2B), and also have a lower proportion of double-positive (CD4 +/CD8 +) T cells [40]. These features possibly reflect less thymic education whereby T cells develop their T cell receptors and are trained to recognize foreign antigens while avoiding self-reactivity.

Mice show a substantial, continuous decline in thymic cellularity beginning shortly after birth (Fig. 3), such that at sexual maturity (~ 4 weeks), the thymus is half the size that it was at birth [40]. Thymic immunosenescence and involution, as previously reported for both humans and mice [67], continues as the mice age, with a reduction in cell number, decreased distinction of cortical-medullary junctions, an increase in fibroblasts and perivascular spaces, and adipocyte (fat) infiltration. Although there was considerable variability among individuals, with a couple of individuals showing few signs of thymic involution, generally definitive signs of thymic involution were evident (Fig. 3) in NMRs, including decreased small lymphocyte (T cell) density, attenuated cortices, increased incidence of cysts, and infiltration of thymic tissue by adipocytes [40]. This observation is at variance with findings previously reported [38]. When comparing the thymi of 3-year-old (*n* = 3) and 11-year-old (*n* = 3) NMRs, Emmrich reported that NMRs did not exhibit thymic involution. One possible reason for the discrepancy between studies may be that the most notable changes in thymic mass occurred during the first year of NMR life (Fig. 3A). Thereafter, the thymic size (both in absolute terms and relative to body size) is comparatively unchanged. However, Lin et al. (2024) report that even after the first year of life, there were also numerous age-dependent histological signs of thymic immune senescence (Fig. 3B and C) with a decline in cortical tissue and cell density, as well as an increase in fat infiltration and large cysts, especially in most of the middle-aged NMRs [37].

### Spleen

**Fig. 4:**
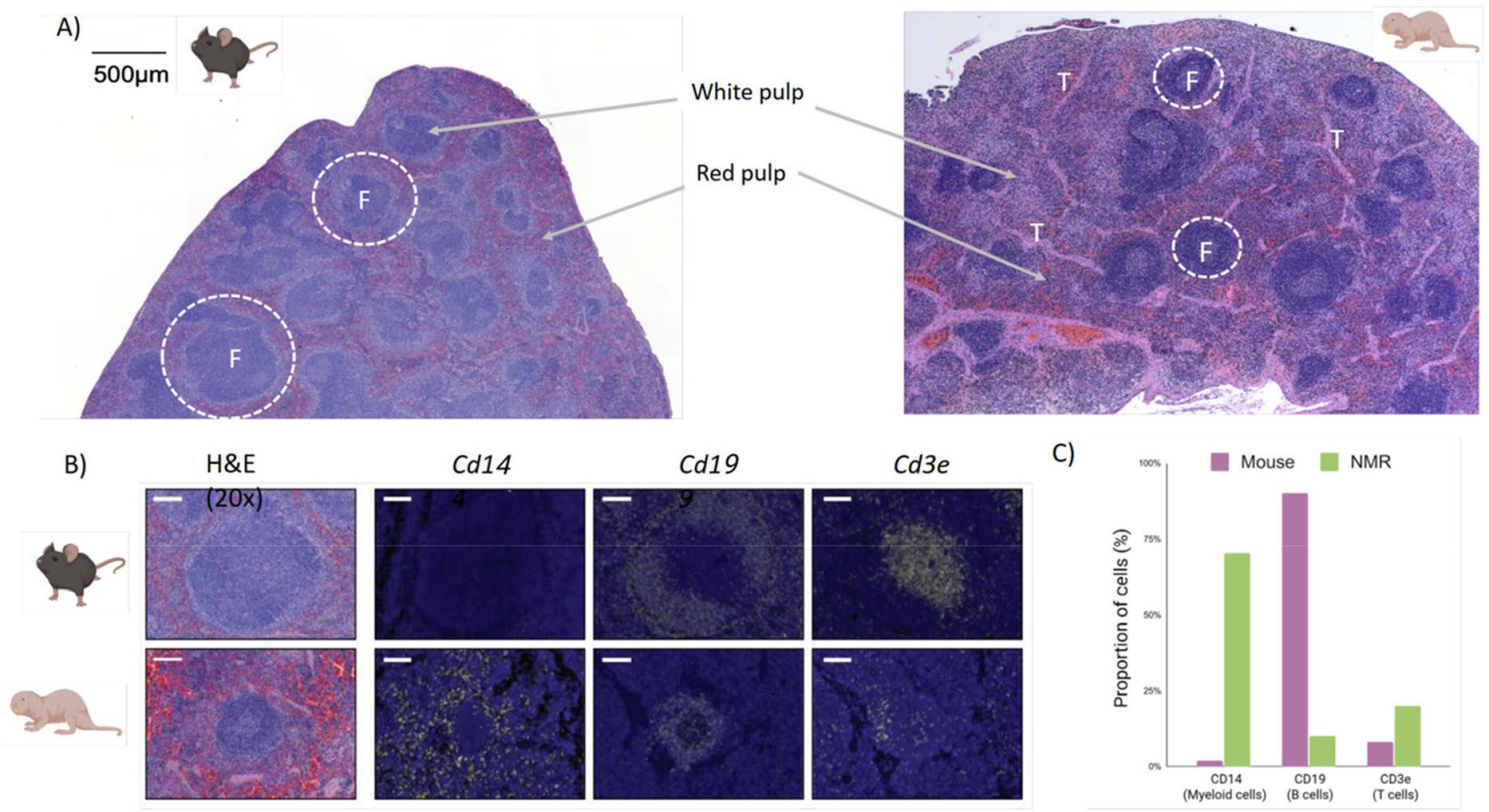
Spleen tissue of young healthy mice and naked mole-rats (NMRs). **A** NMRs have less white pulp as indicated by fewer and smaller follicles. **B** Fluorescence in situ hybridization reveals that the NMR has fewer B (Cd19 +) and T cells (Cd3e +) in the follicles. Myeloid cells (Cd14 +) dominate the red pulp, although T cells are also present in the red pulp, albeit in lower numbers. F = follicles and T = trabecula. **C** The relative proportion of myeloid, B cells, and T cells in splenic tissue. Figure modified from [37] and drawn with BioRender

Spleen mass in healthy NMRs is smaller than that of mice and quite variable in size [39, 68]. Intriguingly, Begay reported that the dominant breeding NMRs had markedly larger spleens than subordinates [39]. Even among subordinates, the spleen size of apparently healthy animals May vary more than fourfold in size, accounting for 0.1–0.35% of body Mass. In sick animals with infections, spleen size May account for 0.2–2.0% of body mass. NMR spleens are more irregularly shaped, slender, and have a paler, purple shade than those of mice. Significant differences in splenic morphology are evident between mice and NMRs (Fig. 4). The NMR has fewer and smaller white pulp follicles (Fig. 4A), with a reduced marginal zone of CD19 + B cells and smaller regions of centralized CD3 + T cells (Fig. 4A). However, these T cells are also found in the red pulp [37, 39]. The larger red pulp component is traversed by numerous trabeculae that provide both structural support and contractility to the spleen. Mice had a smaller red pulp region with smaller and fewer trabeculae and less myeloid (CD14 +) cells (Fig. 4B and C). The larger red pulp and splenic marginal zones of the NMR are rich in pro-inflammatory, cytokine-expressing macrophages and may enable the NMR to mount a rapid and more effective innate immune response [39, 68, 69].

In addition to bone marrow, the spleen is also a major site of hematopoiesis. Splenic extramedullary hematopoiesis in healthy adult NMRs includes erythropoiesis, megakaryopoiesis, and myelopoiesis and appears to occur even under normal physiological conditions. Unlike most mammals, where RBC formation primarily occurs in bone marrow, splenic erythropoiesis in NMRs appears to be yet another neotenic trait in their repertoire of retaining juvenile and fetal traits throughout life [64, 65]. The maintenance of splenic erythropoiesis, in addition to that of bone marrow, may be an adaptation to the hypoxic conditions encountered in deep underground nests. [37, 39]. NMRs have increased extramedullary hematopoiesis in the spleen as they age, and this may contribute to their extreme longevity by preventing age-related anemia.

### Lymph node

NMR axillary and mesenteric lymph nodes are comparable in size to those of mice, measuring 2–4 mm in length [39]. These lymph nodes are organized with typical morphology, containing T cell areas expressing CD3e and B cell areas with germinal centers that are smaller in area compared to those in mice (Fig. 2A, B) [40].

A morphological species-specific difference was observed in the gut-associated lymphoid tissues, most notably with respect to the absence of Peyer’s patches. Peyer’s patches, aggregated lymphoid follicles located primarily in the ileum of the small intestine, are a key component of the gut-associated lymphoid tissue (GALT) in many mammals [70]. They play an essential role in immune surveillance by monitoring intestinal microbial populations and initiating immune responses against pathogens. In contrast to mice, the small intestine of NMRs lacks typical Peyer’s patches, yet their lymphoid nodules contain germinal centers that exhibit significant populations of apoptotic cells [39]. In both species, these T cell areas express CD3e. [39]. The functional implications of these species-specific differences in gut-associated lymphoid tissue remain to be elucidated.

## Immune cells

**Fig. 5:**
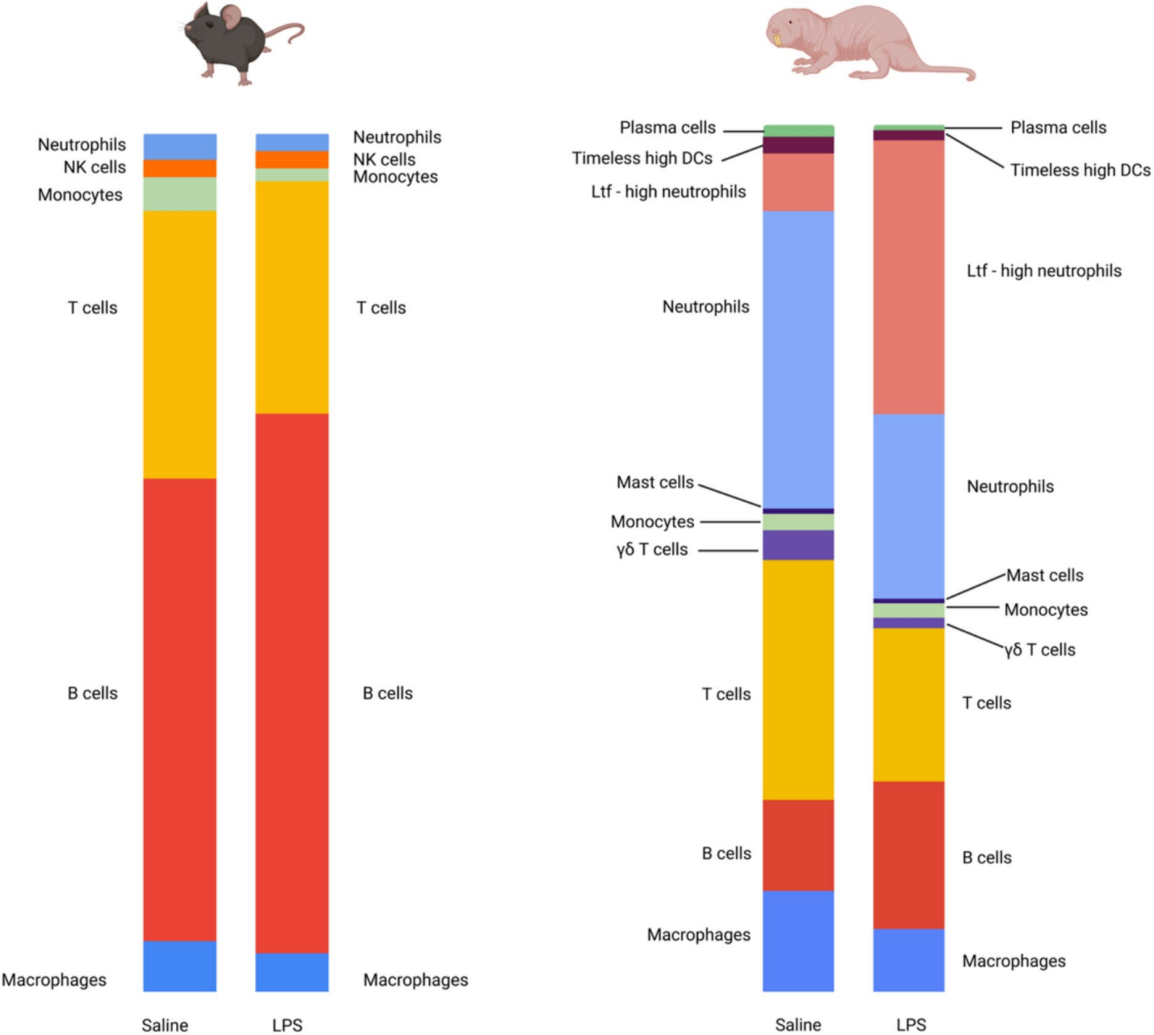
The relative proportion of splenic immune cells in mice and NMRs and the change in population four hours after treatment with lipopolysaccharide (LPS). Erythroid lineage cells are not included. Figure modified from Hilton et al. [37] and Lin et al. [40] and created with BioRender

Using standard, complete blood count (CBC) tests, mice and NMRs have similar total white blood cell counts. All known mouse hematopoietic cells were present in the NMR [39]. However, the relative proportions of the various cell types differed considerably, with the predominant cell type of NMR blood being lymphocytes, followed by neutrophils (Table 1). Immunohistochemical (ICH) and fluorescence in situ hybridization (ISH) studies of spleen tissue revealed that lymphoid cells accounted for less than half of the splenic immune cell population (~ 44%), whereas in mice, lymphoid cells made up most (90%) of the splenic immune cell population (Figs. 4 and 5) [37]. The markedly higher myeloid-to-lymphoid ratio in the blood and spleen of both captive and wild-caught NMRs is more similar to that seen in humans rather than laboratory rodents [39, 71, 72]. Intriguingly, wild-caught mice also show a larger myeloid to lymphoid ratio when compared to laboratory rodents, but their blood has a different immune composition dominated by both neutrophils and B cells [73]. These findings suggest NMRs naturally rely more heavily on myeloid-dependent innate immunity with a more restricted lymphoid-based adaptive immunity.

**Table 1:** White blood cell counts for mice and naked mole-rats (NMRs); unpublished data using cardiac exsanguinated blood and an Abaxis VetScan hematology analyzer

Blood cell (10^9^/l)	Mice		NMR	
	Male (*n* = 9)	Female (*n* = 5)	Male (*n* = 54)	Female (*n* = 42)
Total WBC	7.00 ± 2.59	5.24 ± 2.57	5.91 ± 3.19	5.29 ± 2.37
Lymphocytes	5.31 ± 2.23	3.89 ± 1.70	2.90 ± 1.64	3.15 ± 1.77
Monocytes	0.42 ± 0.37	0.29 ± 0.12	0.33 ± 0.23	0.23 ± 0.16
Neutrophils	1.22 ± 0.86	1.02 ± 1.11	2.62 ± 2.13	1.90 ± 1.12
Eosinophils	0.14 ± 0.08	0.14 ± 0.05	0.08 ± 0.09	0.06 ± 0.02
Basophils	0.04 + 0.02	0.04 ± 0.02	0.03 ± 0.03	0.02 ± 0.01

This NMR myeloid bias is attributed to a large neutrophil fraction, the striking absence of natural killer (NK) cells, and a substantial decline in B cells, as observed using fluorescence ISH and single-cell RNA seq [37]. B cells comprise 10–15% of the total splenic immune cell population, in contrast to mice, where 60% of the splenic immune cells are B cells [37]. The relative proportions of T cells in the splenic immune cell population are similar in mice and NMRs, although the subsets of T cells differed markedly between mice and NMRs [37, 39]. This bias, as observed in young, healthy NMRs and young humans, may reflect that myeloid-biased HSCs have a more persistent capability for self-renewal [74]. Humans, like mice, show an increased myeloid bias with age. This is attributed to a decline in lymphopoiesis, alterations in the HSC population, and the transcriptional up-regulation of genes that specify myeloid-lineage differentiation [75, 76]. This age-related change is thought to be due to a decline in the HSCs (Hoxb5 pHSCs) as well as thymic and splenic dysfunction and the concomitant inhibition of lymphocyte maturation outside of the bone marrow [77]. As a result, aged mice and elderly humans have a less efficient adaptive immune response and a higher incidence of age-related chronic inflammation. In contrast to both mice and humans, the NMR myeloid-to-lymphoid ratio was also observed in bone marrow and did not change significantly with age [40].

### Hematopoietic stem cells

The NMR HSCs are responsible for producing all the blood cell types and produce more erythroid and myeloid lineage cells than lymphoid cells. NMR HSCs are primarily in a quiescent state and exhibit a slower cell cycle, dividing less frequently than mouse HSCs [36]. This prolonged cell cycle reduces the risk of mutation accumulation and damage associated with cell division as well as attenuates stem cell exhaustion—features that may contribute to a stable HSC pool and overall resistance to age-related declines. Unlike mice, NMR HSCs maintain their polarity with advancing age, facilitating both their continued self-renewal and differentiation. Indeed, changes in HSC polarity are commonly linked to age-related decline in function and disease states [78]. Moreover, the HSCs of older NMRs show no increase in inflammatory markers such as interferon (IFN) signaling [79], attenuating another key hallmark of aging [45]. As such, the unusual characteristics of NMR HSCs contribute to the maintenance of a youthful blood cell composition and enhanced protection from cancer and other age-related diseases.

### Erythroid cells

Erythroid lineage cells -such as RBCs and platelets-, like those of immune cells, arise from multipotent HSCs in the bone marrow. NMR megakaryocyte erythroid progenitors (MEPs) present in bone marrow, maintain expression of CD34—a marker commonly associated with less differentiated hematopoietic stem cells—and likely contribute to the significantly lower platelet count and larger myeloid compared to other lab rodents. Moreover, NMRs show no age-associated increase in platelet number, features that may contribute to reduced age-associated chronic inflammation, delayed thrombosis, and, as a result, resistance to age-associated inflammaging-related diseases and their extreme longevity [36, 39]. Platelets are known to contribute substantially to innate immune functions. They are able to mount a rapid response to pathogen-associated molecular patterns (PAMPS) and release bioactive inflammatory molecules that direct other innate and adaptive immune cells to where they are needed and modulate their activity [80, 81]. Further research is needed to specifically understand the role of NMR platelets in their immune function and the implications thereof for their extraordinary longevity and cancer resistance.

### Myeloid immune cells

#### Macrophages

**Fig. 6:**
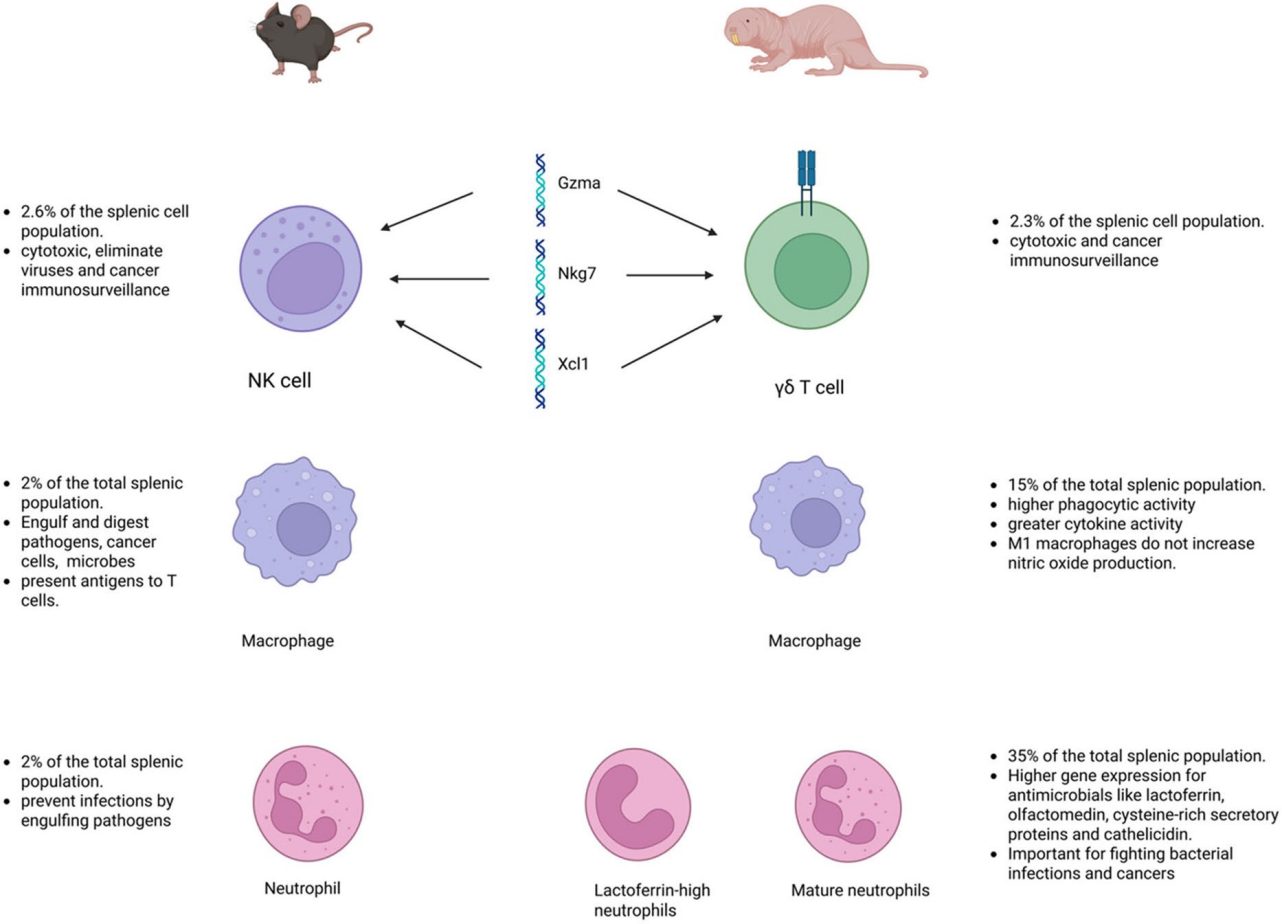
Key immune cells and their function in healthy young mice and naked mole-rats with representative proportions of each cell type. Data were obtained from Hilton et al.[37] and Lin et al. [40] and created with BioRender

Macrophages are a diverse and multifunctional population of mononuclear myeloid cells that play a crucial role in the innate immune system, engulfing and destroying invading bacteria, viruses, cell debris, and foreign bodies, and are essential for wound repair, the activation and resolution of inflammation, and the maintenance of tissue homeostasis. They also mediate additional immune responses by producing signaling molecules (e.g., cytokines), thereby recruiting other immune cells to the site of infection or injury, promoting antigen presentation to T cells, and regulating the adaptive immune response. Approximately 15% of the total NMR splenic immune cell population is macrophages (Fig. 6). These cells have a higher phagocytic capability and greater cytokine production when compared to mouse macrophages [82]. Many naive NMR macrophages also express genes more commonly found in NK cells [82]. Collectively, these NMR traits likely contribute to their greater resistance to foreign substances as well as tumor formation. In response to experimental viral (poly I:C) and bacterial (LPS) stimulation, NMR macrophages, when compared to those of mice, show an attenuated Toll-like receptor (TLR) activation, yet despite this minimal TLR activation, they nevertheless produce higher levels of NFkB and cytokine expression [68]. In addition, under these proinflammatory conditions, NMR macrophages reprogram their metabolism to the expected M1 proinflammatory phenotype, producing both reactive oxygen species (ROS) and proinflammatory cytokines [82, 83]. Unlike mice, they do not increase nitric oxide production [83]. Collectively, these findings suggest that NMR macrophages do reprogram under polarizing stimuli, albeit in a less pronounced and species-specific manner [83]. Although the classification of M1 and M2 macrophages is oversimplified, complicated by their numerous subsets and overlapping phenotypes, it is generally agreed that M2 macrophages help to resolve inflammation and promote tissue repair [84]. Compared to mouse macrophages, those of NMRs show impaired M2 polarization, with unchanged levels of classic M2-associated genes (*Agr1, Egr2,* and *Mrc1*) upon IL-4 stimulation and similarly unchanged OXPHOS and glycolytic metabolism—more in keeping with quiescent cells [83]. This may contribute to the observed longer time intervals to return to baseline levels after an acute proinflammatory procedure [85].

#### Neutrophils

Most myeloid lineage cells present in the NMR spleen are neutrophils transcriptionally recognizable as CXCR2 + granulocytes [37]. Single-cell transcriptomic studies—albeit not the gold standard for the identification of immune cells- suggest that a significant portion of the neutrophils present in bone marrow, spleen, and blood likely are metamyelocytes or immature neutrophils [37]. These immature granulocytes show higher levels of gene expression for antimicrobials, such as lactoferrin, olfactomedin, cysteine-rich secretory proteins, and cathelicidin (Fig. 6), and account for ~ 1–5% of the entire NMR splenic cell population [37]. These “lactoferrin-high” neutrophils are rapidly activated in response to LPS stimulation and are accompanied by the activation of numerous inflammatory pathways [37]. In healthy humans and mice, only mature neutrophils are present in peripheral blood, with immature granulocytes typically found in bone marrow and released into the bloodstream when severe infections are encountered [86]. It is tempting to speculate that consistently high levels of lactoferrin-high, LPS-responsive granulocytes in healthy NMRs may indicate that NMRs maintain an “activated or primed” state to more rapidly and efficiently respond to infections. Further histological and cell sorting studies (such as Max-Grunwald staining) are needed to confirm that these lactoferrin-high cells are indeed immature neutrophils present in the spleens and peripheral blood of healthy NMRs. The high levels of the antimicrobials (lactoferrin, cathelicidin, and olfactomedin) in this lactoferrin-high neutrophil population may also confer cancer resistance by inhibiting tumor growth, arresting the cell cycle of cancer cells, and promoting programmed cell death through iron-mediated cell membrane disruption [87–89]. Intriguingly, a higher proportion of lactoferrin-high neutrophils is reportedly present in the larger spleens of dominant NMRs [39] and may contribute to their considerably longer lifespans than subordinates [28]. it is tempting to postulate that the disparate proportion of splenic immune cells, as well as their larger spleens, affords some survival advantage to the breeding females, protecting them from infections arising while fighting to maintain dominance as well as facilitating better tissue repair.

### Lymphoid immune cells

#### Natural killer cells and natural killer T cells

Apart from the disparate proportion of myeloid and lymphoid cells between mice and NMRs, the most striking interspecies difference was the lack of canonical NK-cells in NMRs (Fig. 6). Indeed, no Cd3 or Cd8 negative cells were expressing the classic NK-cell markers of Gzma, Gzmb, Klrd1, and Ncr1 in either the spleen or circulating peripheral blood [37, 69]. The lack of NK cells is further supported by a dearth of genes that control NK function, most notably the genes encoding NK cell receptors and their major histocompatibility class I (MHC-I) ligands. In other species, NK cells also play a crucial role in defending the body against viral infections, an essential component of the innate immune system. In this regard, they produce cytokines (e.g., TNFa, IFNg, IL10), secrete cytotoxic granules, and employ death receptor-mediated cytolysis. The absence of NK cells in NMRs may be due to the relaxation of viral selection pressures. These animals live in a cloistered underground habitat sealed off from airborne pathogens and contact with animals outside their family group. As a result of this absence of selective pressure, inactivating mutations in both NK cell genes and the genes that regulate NK cell function (e.g., MHC-I and NK cell receptor genes [Lyr 49 genes]) may accumulate. Over time, both pseudogenes as well as the total loss of NK-related genes may arise [37, 69]. Given the importance of NK cells as the first line of defense against viral pathogens, it is not surprising that in captivity, NMRs are reportedly highly susceptible to viral infections such as those from Coronavirus and Herpes virus [90, 91], with mass die-off events evident due to both known and unknown causes [22, 39, 90–92]. The low IFN-γ signaling, coupled with the absence of these cytotoxic cells, may contribute to an inadequate anti-viral response of NMRs. Susceptibility to viral pathogens is exacerbated further by the microarchitecture of the spleen, with reduced white pulp, a reduced marginal zone area, and concomitant low numbers of B cell lineages, and a small marginal zone macrophage population [37, 39], suggestive of impaired clearance of blood-borne pathogens and limited pathogen memory. Since NK cells are crucial for immune surveillance, patrolling the body and assisting in the rapid identification and eradication of abnormal cells without the need for prior sensitization or activation, their absence also could result in worse cancer immunosurveillance, unless other cells take up that function and compensate for their loss.

Natural killer T (NKT) cells are unique lymphocytes that bridge the innate and adaptive immune systems; these cells reportedly rely on Cluster of Differentiation 1 (CD1) proteins for their development [93]. It appears that the NMR CD1b is dysfunctional, and CD1d and CD1e have been lost, collectively contributing to a loss of CD1d/NKT cells in the NMR immune repertoire [94]. This loss likely also contributes to the heightened susceptibility of captive NMR populations to viral infections. Furthermore, the absence of NK and CD1d/NKT cells may lead to reduced levels of IFN-γ and TNF-α, thereby attenuating the secretion of other proinflammatory cytokines, such as IL-6 and CXCL8, by keratinocytes [95]. This attenuation likely underlies the observed lack of psoriasis-like skin inflammation in NMRs treated with imiquimod, a Toll-like receptor (TLR) 7/8 agonist that typically induces epidermal thickening, parakeratosis, and leukocyte infiltration in other species [94, 96]. The precise mechanisms by which these immune cell deficiencies modulate inflammatory responses remain elusive.

#### B cells

B cells play a critical role in adaptive immunity. These cells secrete cytokines that help coordinate and regulate the intensity of the humoral immune response [70]. They also produce antibodies that bind to specific antigens and act as antigen-presenting cells (APCs), thereby assisting T cell recognition of the pathogen and initiating the targeting of pathogens for destruction. The B cell compartment is markedly reduced in NMRs (Figs. 4 and 5) with a strikingly small marginal zone area, and the gene sets involved in B cell proliferation, regulation, and homeostasis are also underrepresented [37, 39]. Very little is known about NMR B cells since currently there are very few reagents that can be reliably used in NMRs to study their functions. The reduced B cell component of NMRs, as indicated by transcriptomics and fluorescence-ISH, likely reflects a shift in the immune repertoire away from humoral immunity towards T cell–driven immune surveillance.

#### T cells

CD8 and CD4 T cells eliminate intracellular and extracellular infectious agents, relying upon T cell receptor (TCR) recognition of peptides presented upon MHC I and MHC II, respectively. NMR spleens have a low CD8/CD4 *αβ* T cell ratio [40]. The enrichment of CD4 + over CD8 + cells in NMRs may indicate that NMRs show attenuated cell-mediated immunity or possibly that antigen presentation from CD4 to B cells is more efficient than that seen in other mammals.

The low CD8/CD4 *αβ* T cell ratio is accompanied by lower clonotypic diversity than seen in mice [40, 41]. During genomic rearrangement, some T cells are committed to the γδ lineage [97] and their γδ TCRs recognize both self and foreign stress ligands [98]. These cells are the first to emerge in human and mouse embryonic thymi and mainly populate peripheral tissues such as skin and gut [98, 99]. γδ T cells are preprogrammed during development and present in both the NMR ectopic and thoracic thymic tissue and the bone marrow. Unlike mice, NMRs γδ T cells lack the expression of *Itgae*, a gene that encodes an integrin that directs the mouse and human γδ T cells to their resident epithelial target tissues upon their egress from the thymus [40]. This likely contributes to the large splenic and circulating γδ T cell population of NMRs (Figs. 5 and 6).

The NMR genome encodes a large variety of* γ* and *δ* variable TCR regions; this is striking when compared to the far smaller diversity of the *α* and* β* variable TCR regions. The absence of Cd8*β* expression implies that Cd8*α* and Cd8*β* heterodimers cannot form, preventing *αβ-*TCR-MHC-I binding and the subsequent classical Cd8-T cell activation, leading to a decrease in TCR sensitivity to MHC-I. Rather, Cd8-T cell activation likely occurs via non-classical MHC-I ligands associated with the large clonotypic diversity of γδ T cells. These are likely capable of recognizing an extensive range of non-MHC-I ligands. Although not present in mice and humans, a large circulating γδ T cell population, with a wide range of genomic diversity of *γ* and *δ* variable TCR regions, is not unique to NMRs, but is also found in pigs, horses, and ruminants [100, 101]. These γδ T cells are thought to increase the T cell compartment’s spatial and temporal responsiveness, contributing to both innate and adaptive immunity [102]. Intriguingly, in human patients with a diverse suite of tumor types, those with a larger fraction of resident γδ T cells reportedly had a more favorable cancer prognosis. It is possible that having a large circulating population of γδ T cells facilitates more efficient tumor immunosurveillance and concomitant elimination of senescent cells and may be an intrinsic component of their prolonged good health and resistance to age-related chronic diseases.

The γδ T cell population occupies a similar splenic proportion to mouse splenic NK cells (Fig. 5). It is highly oligoclonal with two distinct γδ TCR populations, including one with cytotoxic capabilities that harbors a distinct dominant invariant clonotype [40, 41]. *Gzma*, *Nkg7*, and *Xcl1* cytotoxic genes, are typically expressed in both the mouse NK cells and the NMR γδ T cell population, suggesting that these two cell types are functionally similar. However, they are likely subject to different mechanisms of activation. While Cd8 *αβ* T cells require peripheral activation for effector cell differentiation, these γδ T cells are likely developmentally programmed within the thymus to have distinct molecular signatures associated with different effector subsets [38]. The second distinct γδ T cell cluster population appears more variable. It appears to have different functions, including the expression of type-3 immunity-associated genes related to IL-17 production and enrichment with molecules of the TNF receptor family. This larger clonotypic diversity of this second population cluster of γδ T cells may have evolved to recognize a broad variety of non-MHC-I ligands. As such, these cells may be more instrumental in eliminating early malignancies, senescent and/or damaged cells, thereby efficiently maintaining tissue homeostasis.

Collectively, these findings regarding T cell populations suggest that NMRs evolved under relaxed pathogenic selective pressures over the course of evolution, with a stronger target for selection being firstly the maintenance of undamaged youthful cells and secondly cancer immunosurveillance, with the concomitant result of cancer resistance, prolonged good health, and enhanced longevity.

## Responses to extracellular pathogens

The reported greater susceptibility of NMRs to viral [39, 90, 91] and parasitic (e.g., *Leishmania donovani*) infections [103] may be due to the reductions in lymphoid lineage and reduced IFN signaling. In addition, reduced numbers of marginal zone macrophages and dendritic cells, in addition to the complete lack of NK cells [37], may result in an attenuated response to counter viral infections. Better protection against bacterial and fungal infections may be evident, likely due to the larger population of γδ T cells [99, 104] and the potentially primed LPS-responsive lactoferrin-high neutrophil population [37]. While naturally occurring bacterial and fungal infections are relatively rare in both zoos and research facilities, purportedly due to strict hygiene practices, many animals injured during fighting do develop purulent secondary abscesses, most notably caused by infection with *Staphylococcus* species from which they often are unable to recover, without antibiotic intervention (personal observations; [22]). Similarly, mimicking acute bacterial infections by treating animals with lipopolysaccharide (LPS), induced pronounced immune responses in both mice and NMRs with marked changes in both lymphoid and myeloid immune cells (Fig. 5). Both species activated similar pathways, although the responses were markedly less pronounced in the NMR [15, 37].

## Naked mole-rat responses to intracellular stressors and age-related diseases

Non-infectious degenerative changes, including hepatic and myocardial megalocytosis, hepatic hemosiderosis, and lipofuscin accumulation, may occur spontaneously in NMRs and are often associated with advancing age – being more common in 10–20 year old animals [22]. Although extremely rare, myocarditis—inflammation of the heart resulting in injury of cardiac myocytes—has been observed in NMRs with an unknown etiology. A constellation of degenerative changes in the NMR kidney referred to as chronic progressive nephropathy (CPN) has been commonly diagnosed in middle-aged and older adult NMRs. CPN in NMRs has an unknown pathogenesis but has lesions similar to those described in lab rats and mice [17] and includes lesions in tubules (ectasia, proteinosis, degeneration, necrosis, atrophy, drop out), interstitial aggregates of lymphocytes, plasma cells, and macrophages (nephritis), and to a lesser extent membranous glomerulopathy or sclerosis [22].

Spontaneous neoplasms have been reported in NMRs in both zoo and research settings, albeit in extremely low numbers relative to other species [17, 22, 105, 106]. Malignant masses arising from epithelial cells (i.e., carcinomas) have been the most frequently diagnosed: these include mammary adenocarcinoma, hepatocellular carcinoma, and esophageal carcinoma. Neuroendocrine tumors in the thyroid and stomach have also been reported [106]. While lymphoma, the neoplastic proliferation of lymphocytes or lymphoid progenitors, is very common in humans and laboratory rodents, there has only been a single case of disseminated T cell lymphoma in a euthanized 26-year-old breeding female [22]. In most other cases of proliferation of lymphocytes in the spleen, thymus, or lymph nodes, no signs of dissemination to other lymphoid tissues have been observed. Pre-neoplastic lesions based on histologic characteristics defined in lab mice and rats [107] have been observed more commonly [17, 22]. In most species, tumorigenesis is attributed to the accumulation of DNA mutations in response to DNA-damaging agents and the resulting proinflammatory microenvironment, with the accrual of DNA damage and associated inflammation closely Linked to age-related functional declines, the development of chronic diseases such as cancer, and species Lifespan. NMRs are resistant to experimental carcinogenesis using both 3MC and DMBA/TPA chemical carcinogens [15] and regular exposure to high levels of UV radiation (unpublished data). This inability to experimentally induce tumor formation is partially attributed to an attenuated inflammatory response to carcinogen-induced tissue damage as well as reduced accumulation of somatic DNA mutations.

Recent studies have directly measured somatic mutation rates in NMRs. Analysis of whole-genome sequences of intestinal crypts from various mammalian species, including NMRs, found that somatic mutation rates per year are inversely correlated with lifespan [108]. Specifically, NMRs, with their extended Lifespan of up to 40 years, exhibit lower annual somatic mutation rates compared to shorter-lived species like mice. Interestingly, it was found that the NMR was the biggest outlier to the inverse relationship between body mass and annual somatic mutation rate. This suggests that NMRs employ unique mechanisms that defy the traditional allometric scaling laws to accumulate fewer mutations per unit time, contributing to their cancer resistance and longevity.

Furthermore, NMRs possess enhanced DNA repair mechanisms. When the liver transcriptomes of NMRs, humans, and mice were compared, it was revealed that NMRs have significantly higher expression of DNA repair genes [109]. Specifically, 34 DNA repair genes showed more than a two-fold higher expression in NMRs compared to mice, including TP53, MSH5, Ku70, and several DNA polymerases. Significant upregulation of DNA repair pathways, including mismatch repair, non-homologous end-joining, and DNA damage response signaling, compared to those in mouse, was observed. This enhanced DNA repair capacity likely plays a crucial role in maintaining genomic stability and preventing the accumulation of mutations. Further studies have elucidated these transcriptional changes and demonstrated the mechanistic or phenotypic effects of the enhanced DNA repair in NMRs. SIRT6 facilitates more efficient DNA double-strand break repair in long-lived species, including NMRs, where elevated. SIRT6 activity was found with coincident enhanced PARP1 function, thereby improving DNA repair processes [109–111]. Another important feature of the DNA repair pathway, excision repair, demonstrated that NMR cell extracts are 2–3 times more efficient at removing bulky DNA lesions and 1.5–3 times more efficient at removing uracil compared to mouse cells, indicating superior base excision repair (BER) and nucleotide excision repair (NER) capabilities [112]. Interestingly, while both NMRs and humans show higher DNA repair gene expression than mice, humans have significantly higher expression in the BER pathway compared to NMRs and mice, whereas NMRs exhibit unique upregulation in mismatch repair and DNA damage response signaling pathways [109]. This unique constellation of DNA repair mechanisms highlights the novel location of the NMR compared to other animal model systems on the high-dimensional fitness landscape, undeniably contributing to their exceptional longevity and cancer resistance.

In addition to efficient DNA repair, NMRs also exhibit resistance to DNA damage-induced cell death. NMR fibroblasts display lower levels of caspase 3/7 activity and require higher concentrations of DNA damage-inducing agents to undergo apoptosis or necroptosis [15]. This resistance is linked to loss-of-function mutations in genes regulating necroptosis, such as RIPK3 and MLKL, which prevent the release of damage-associated molecular patterns (DAMPs) and subsequent inflammation [15]. Consequently, the attenuated inflammatory response and immune cell infiltration reduce the pro-tumorigenic microenvironment, further contributing to cancer resistance [111]. The combination of lower somatic mutation rates, enhanced DNA repair mechanisms, and an attenuated inflammatory response underpins the exceptional resistance of NMRs to cancer and age-related diseases, offering valuable insights into the mechanisms of longevity and genomic stability.

## Age-related changes in the immune system

Significant differences in immune cell populations between neonatal and adult NMRs have been documented [41]. Yet, age-related changes in mature adult NMRs are equivocal as these animals exhibit considerable individual variability, complicating the identification of consistent patterns [40]. Lin et al. (2024) compared immune cell composition in young adult NMRs (2–3 years, *n* = 3) with that in old NMRs (26–28 years, *n* = 4). Notably, only two of the four older NMRs displayed pronounced age-related changes, including a decline in CD8 T cells (from 7 to 2.3%) and an increase in cytotoxic γδ T cells, with innate lymphoid cells (ILCs) detected in two of the four old animals but absent in the young cohort. In contrast, in that same study, old C57BL/6 mice (24 months, *n* = 4,) consistently exhibited a higher proportion of memory T cell subsets (CD8, CD4, and Gzmk-high CD8 lymphoid cells), while young mice (2 months, *n* = 4) had a greater proportion of naïve T cell subsets. Emmrich et al. also reported a lack of age-related Changes in the blood cell types of 3-year-old (*n* = 3) and 11-year-old (*n* = 3) NMRs [36]. The small sample sizes and marked individual variability in NMRs underscore the need for larger studies to elucidate age-related immune changes and their functional implications. NMRs, like mice, generally show signs of thymic involution (Fig. 3) and middle-aged (10–12 years old) NMRs, like mice, exhibit pathologies generally associated with aging, such as renal tubular calcinosis, chronic progressive nephropathy, hepatic hemosiderosis, and lipofuscinosis in tissues like the brain and adrenal glands [12, 17]. These pathological features may be associated with immune alterations: however, further research is required to establish a direct link between these and immune system changes in NMRs.

A widely accepted concept within the field of aging is “inflammaging”—i.e., the continuous and monotonically increasing baseline level of chronic inflammation with advancing years. The NMR may have evolved mechanisms to prevent this inflammaging burden as it has been proposed that the diminished anti-viral response of the NMR, namely the reduction in IFNg, TNFa, and perhaps retrotransposon-mediated proinflammatory signaling, forms a protective component of the NMR’s “negligible-senescence” phenotype [36]. Clearly, larger studies are needed to fully assess age-related changes in the NMR immune system and the functional significance thereof.

## Future directions

The NMR is emerging as a valuable model in biomedical research due to their many unique biological adaptations to a subterranean lifestyle and their exceptional prolonged lifespan and healthspan [113]. Technological advances are providing exciting new tools for molecular assessments in novel species like the NMR, abrogating their reliance on species-specific antibodies, and are already yielding significant breakthroughs in our understanding of aging, hypoxia tolerance, and disease resistance. Many of these new insights have generated more questions than they have answered. For example, aging studies have revealed that dominant females, despite the metabolic and physiological demands of pregnancy and lactation, live considerably longer than subordinates [28]. Moreover, they appear to reset their DNA methylation clock exhibiting a slower rate of epigenetic aging than same age subordinates [[114]. These queens also appear to have disparate spleen sizes and possibly better protective mechanisms within their immune system [39]. Elucidating these immune and aging mechanisms may yield novel therapeutic targets, especially since any female, regardless of age, can usurp dominance and revert to a more youthful phenotype. Genomic analysis of the two published genomes that were built using high coverage Illumina sequencing and sequence homology with human and mouse well-curated genomes were used in NMR gene annotation. Lewis reported that a large component of the NMR genome had escaped annotation and that the only genes known are those that are identified in mice and humans [115]. Clearly, further comprehensive genomic data analyses are needed that take advantage of technological and bioinformatical improvements that yield higher chromosome-scale resolution (e.g., using more recently published genomes) [116–118]. Additional future research should prioritize developing NMR-specific immunological reagents, leveraging single-cell omics for longitudinal aging studies, and conducting in vivo challenge experiments to bridge these knowledge gaps that still exist. There is also a need for follow-up confirmatory studies to unambiguously elucidate those genes and regulatory pathways that are directly pertinent to their unique immune system, disease resistance, and prolonged longevity.

In addition, it is essential to evaluate if there are signs of immune-senescence over the four-decade NMR lifespan. Given the greater reliance on the innate immune system, does it maintain effectiveness in pathogen recognition and clearance? Similarly, do the unusual adaptive immune features become less responsive in elderly animals or are immune-protective mechanisms upregulated? Huge gaps remain in our understanding of the naked mole-rat immune system and there remain many unanswered questions regarding its function in response to viral and bacterial pathogens, in regulating inflammation, and even in dealing with transformed preneoplastic cells or spontaneous cancers should they arise. Similarly, an important unanswered question concerns its role in maintaining a prolonged youthful state, or indeed if it contributes to preventing the seemingly inevitable vagaries of aging.

## Summary

Although NMRs are rodents, they surprisingly show more similarities to young, healthy humans’ immune systems than conventional laboratory rodents. Like humans, their bone marrow, spleen, and blood have a larger proportion of myeloid lineage cells relative to lymphoid cells. This ratio and immune profile are conserved in mice until they are old, at which point they have comparable myeloid-to-lymphoid ratios as well as similar spleen architecture. Why this is the case is currently unknown. The NMR immune repertoire shares other features with humans that are absent in mice; most notably, immature and mature neutrophils are morphologically similar. Moreover, human immune system antibodies show better cross-reactivity with NMRs than mice. The cellular and molecular underpinnings of these features remain elusive. Like humans and mice, thymic involution occurs shortly before puberty. A decline in cell density, coupled with increased fat infiltration and fibrosis, continues to occur as the animals age. To date, most studies have been undertaken in subordinate animals rather than the longer-lived dominant breeders. It is possible that disparate immune composition and function may contribute to the divergent Gompertzian mortality rates of subordinates and dominant animals. In support of this is a report that healthy dominant animals have larger spleens and a larger proportion of lactoferrin-high neutrophils in their peripheral circulation and spleen. This immune cell composition that may better protect against all-cause mortality. Studies of the NMR immune system and its role in health and disease are still in their infancy. Furthermore, in-depth comparative biology studies are essential to better understand the regulatory molecular mechanisms involved and the properties and function of the unusual NMR immune system and its role in defying the vagaries of time.
